# Intervening and Acting With and For: Intervention Research as a Necessary Actor in Cancer Control

**DOI:** 10.17269/s41997-026-01165-2

**Published:** 2026-04-29

**Authors:** Norbert Ifrah

**Affiliations:** 1 Président de l’Institut national du cancer (INCa), 52 avenue André Morizet, Boulogne-Billancourt, 92513 France; 2 President of the French National Cancer Institute (INCa), 52 avenue André Morizet, Boulogne-Billancourt, 92513 France

Norbert Ifrah, Président de l’Institut national du cancer (INCa), 52 avenue André Morizet, Boulogne-Billancourt, 92513, France

*Dans un contexte marqué par l’augmentation des cancers et la persistance des inégalités de santé, la recherche interventionnelle en santé des populations (RISP) est un levier activable dans la lutte contre les cancers, susceptible d’ouvrir la voie à des solutions co-construites, ancrées dans les réalités sociales et territoriales.*

Les données épidémiologiques récentes soulignent l’ampleur et la complexité des défis liés aux cancers en France. En 2023, on estime à 433 136 le nombre de nouveaux cas diagnostiqués et à 164 095 le nombre de décès par cancer sur la même période (Institut national du cancer (INCa), 2025). Si le vieillissement de la population contribue en partie à l’augmentation du nombre de cas, l’impact de certains facteurs de risque – tels que le tabac, l’alcool, l’alimentation ou l’exposition aux rayonnements ultraviolets – demeure prégnant ; ces facteurs étant impliqués dans près de 40 % des cancers évitables (Société Française d’Alcoologie, n. d.).

Ce contexte appelle des stratégies intégrées, qui ne se contentent pas d’agir sur les déterminants biologiques ou comportementaux, mais qui prennent également en compte les environnements sociaux, culturels et économiques dans lesquels vivent les populations. La RISP doit se situer précisément à cette intersection : combiner une compréhension systémique des enjeux de santé avec une capacité d’action concrète, pour réduire l’incidence des cancers, améliorer la qualité de vie des personnes concernées et diminuer les inégalités sociales et territoriales face à la maladie.

Depuis plus d’une décennie, l’Institut national du cancer (INCa) soutient contre vents et marées la recherche interventionnelle en santé des populations. Celle-ci ne saurait se limiter à décrire des phénomènes de santé ou à identifier des déterminants : sa mission est de concevoir, mettre en œuvre et évaluer des interventions efficaces, contextualisées et reproductibles, susceptibles de modifier concrètement les pratiques, les politiques et les trajectoires de santé (ERA4Health Partnership, n.d.). C’est en ce sens qu’elle peut devenir une « science des solutions », titre que la chimie pourrait aussi revendiquer…

S’appuyant sur des approches méthodologiques diversifiées, souvent pluridisciplinaires, la RISP doit mobiliser et articuler des savoirs issus d’horizons multiples : chercheurs, professionnels de santé, décideurs, patients, représentants d’usagers, aidants et acteurs de terrain. Elle suppose une dynamique de co-construction des interventions, pensées et déployées pour et avec les populations, quelles que soient leurs fragilités ou leurs vulnérabilités, afin de garantir leur pertinence, leur acceptabilité et leur transférabilité.

Appliqué au cancer, le champ de la RISP est celui du développement des interventions et des dispositifs couvrant l’ensemble du continuum de la maladie : de la prévention primaire au dépistage précoce, en passant par la prévention quaternaire, avec une attention particulière portée à la réduction des inégalités de santé.

L’INCa a joué, et continue de jouer, un rôle moteur dans le développement et la structuration de la recherche interventionnelle. Depuis 2010, plus de 23 millions d’euros ont été engagés pour soutenir la RISP appliquée au cancer à travers différents dispositifs, contribuant ainsi à améliorer les connaissances et à éprouver des dispositifs de recherche en prévention dont certains sont aujourd’hui déployés en actions de terrain, tels que Tabado.^1^

L’INCa est l’un des principaux acteurs publics engagés dans le financement et le déploiement de la RISP et, plus largement, de la recherche en prévention, avec une volonté affirmée d’en renforcer le rayonnement. Aussi, son action s’inscrit dans un écosystème national structuré, dans lequel différents acteurs travaillent de concert – tels que l’Institut pour la recherche en santé publique (Hawe & Potvin, 2009), l’Association française d’alcoologie (Institut pour la Recherche en Santé Publique (IReSP), n. d.), l’Agence nationale de la recherche (Les cancers, 2018) ou encore l’Union européenne (Agence Nationale de la Recherche (ANR), n. d.) – contribuant ainsi collectivement à la cohérence et à la portée globale de la recherche en prévention en France. Ainsi, plusieurs facteurs de risque de cancers étant partagés avec d’autres maladies, par exemple les maladies cardiovasculaires pour le tabac et l’alcool, les efforts de recherche en prévention de ces facteurs financés dans d’autres domaines que les cancers par d’autres organismes s’ajoutent à ceux de l’INCa. Par ailleurs, il faut ajouter à ces efforts de financement les soutiens nationaux pour les infrastructures et les personnels de la recherche publique qui n’apparaissent pas dans les budgets attribués par les organismes de financement.

La mise en œuvre de la Stratégie décennale de lutte contre les cancers 2021-2030 (Institut national du cancer (INCa), 2021) a particulièrement contribué à renforcer le déploiement de la RISP par des actions et des enjeux concrets, pour lesquels sa contribution et, plus largement, celle des sciences humaines et sociales sont attendues, telles que : les approches personnalisées de prévention, l’amélioration de l’accès aux dépistages, la promotion de comportements favorables à la santé ou encore le développement des soins de support, etc.

Parallèlement, la participation des citoyens, des patients, des travailleurs et, plus largement, des bénéficiaires des interventions s’est diffusée et valorisée dans les discours scientifiques et institutionnels. Sur cette question, l’INCa a d’ailleurs lancé un comité de démocratie sanitaire. Cependant, dans les espaces de recherche, la participation des parties prenantes reste un enjeu tant sur le plan méthodologique qu’éthique et organisationnel. C’est dans ce contexte que l’INCa a organisé, les 12 et 13 décembre 2023, le 6ᵉ colloque international francophone intitulé : « Intervenir, agir avec et pour les citoyens, patients, travailleurs : apports dans le champ du cancer de la recherche interventionnelle en santé des populations ». 

Les contributions réunies dans ce supplément interrogent ces enjeux avec rigueur et nuance. Elles montrent que le « faire avec et pour » ne se réduit pas à un principe méthodologique, mais implique une redéfinition des relations entre chercheurs, professionnels, acteurs de terrain, patients et citoyens. Cette dynamique participative invite à dépasser les cloisonnements traditionnels entre champs disciplinaires, milieux de vie et statuts ; le patient étant aussi un citoyen et un travailleur, et inversement.

Un premier ensemble de contributions explore les fondements conceptuels, méthodologiques et théoriques de la recherche interventionnelle, en mettant l’accent sur la co-construction des interventions, la capitalisation des connaissances et les cadres permettant de penser l’évaluation et la transférabilité des actions (Villeval et al. ; Beuriot et al.).

Un second axe porte sur les interventions de prévention et de détection précoce des cancers, notamment en milieu professionnel ou auprès de populations spécifiques (Rollin et al. ; Crasset et al. ; Hunsmann et al.). D’autres contributions mettent en avant les démarches participatives et inclusives, en s’intéressant à l’implication des patients, du public ou de groupes vulnérables dans la production de connaissances et l’apprentissage en santé (Bauquier et al. ; Oustric et al. ; Satgé et al.).

Un second axe porte sur les interventions de prévention et de détection précoce des cancers, notamment en milieu professionnel ou auprès de populations spécifiques (Rollin et al.; Crasset et al.; Hunsmann et al.).

D’autres contributions mettent en avant les démarches participatives et inclusives, en s’intéressant à l’implication des patients, du public ou de groupes vulnérables dans la production de connaissances et l’apprentissage en santé (Bauquier et al.; Oustric et al.; Satgé et al.).

Enfin, un quatrième axe interroge les conditions d’implémentation, de mise à l’échelle et de pérennisation d’interventions complexes, qu’elles concernent l’arrêt du tabac, le dépistage, l’amélioration de l’expérience de soins ou l’accompagnement social et économique des personnes en situation de précarité (Stevens et al. ; Burtin et al. ; Pérignon et al. ; Pomey et al.). 

Parce que les défis sanitaires et sociaux se complexifient, la recherche interventionnelle apparaît plus que jamais comme un outil important pour agir avec et pour les populations, au service d’une lutte contre les cancers à la fois efficace, équitable et durable. Mais à un moment de l’histoire où le contexte international impose à chacun de justifier chaque dépense, il est essentiel que chaque domaine de recherche prouve la place qu’il a su prendre dans les avancées collectives : s’agissant, par exemple, de la réduction du tabagisme quotidien, l’analyse – y compris médico-économique – du poids respectif des efforts cognitivo-comportementaux et des interventions réglementaires (prix, interdictions, substitutions) ne saurait être éludée.

Ce supplément vise ainsi à susciter l’intérêt de l’ensemble des acteurs impliqués dans la recherche interventionnelle afin d’encourager le développement de nouvelles collaborations et de nouvelles initiatives.

*In a context marked by the rising incidence of cancer and the persistence of health inequalities, population health intervention research (PHIR) represents a key lever in the fight against cancer. It has the potential to pave the way for co-constructed solutions that are firmly rooted in social and territorial realities.*

Recent epidemiological data highlight the scale and complexity of cancer-related challenges in France. In 2023, an estimated 433,136 new cancer cases were diagnosed, and 164,095 deaths from cancer occurred during the same period (Institut national du cancer (INCa), 2025). While population ageing partly contributes to the increasing number of cases, the impact of certain risk factors—such as tobacco use, alcohol consumption, diet, and exposure to ultraviolet radiation—remains substantial; these factors are involved in nearly 40% of preventable cancers (Société Française d’Alcoologie, n. d.).

This context calls for integrated strategies that go beyond addressing biological or behavioural determinants alone, and that also take into account the social, cultural, and economic environments in which populations live. PHIR is positioned precisely at this intersection: combining a systemic understanding of health issues with concrete action capacity, in order to reduce cancer incidence, improve the quality of life of affected individuals, and decrease social and territorial inequalities in relation to the disease.

For more than a decade, the French National Cancer Institute (INCa) has supported population health intervention research against all odds. This field cannot be limited to describing health phenomena or identifying determinants; its mission is to design, implement, and evaluate effective, context-sensitive, and reproducible interventions capable of producing tangible changes in practices, policies, and health trajectories (ERA4Health Partnership, n. d.). In this sense, PHIR can be seen as a “science of solutions,” a title that chemistry might also lay claim to.

Drawing on diverse and often multidisciplinary methodological approaches, PHIR mobilizes and connects knowledge from multiple backgrounds, including researchers, health professionals, decision-makers, patients, user representatives, caregivers, and community-based actors. It relies on a dynamic of co-construction of interventions that are designed and implemented for and with populations, regardless of their fragilities or vulnerabilities, in order to ensure relevance, acceptability, and transferability.

When applied to cancer, PHIR encompasses the development of interventions and programmes across the entire continuum of the disease—from primary prevention to early detection, including quaternary prevention—with particular attention paid to reducing health inequalities.

INCa has played, and continues to play, a driving role in the development and structuring of intervention research. Since 2010, more than €23 million have been invested to support cancer-related PHIR through various funding schemes, thereby contributing to the advancement of knowledge and to the testing of prevention research interventions, some of which are now implemented in real-world settings, such as the Tabado programme.^2^

INCa is one of the main public actors involved in funding and deploying PHIR and, more broadly, prevention research, with a clear commitment to strengthening its visibility and impact. Its action is embedded within a structured national ecosystem in which various stakeholders work collaboratively—such as the Institute for Public Health Research (Hawe & Potvin, 2009), the French Association of Alcohol Studies (Institut pour la Recherche en Santé Publique (IReSP), n. d.), the National Research Agency (Les cancers, 2018), and the European Union (Agence Nationale de la Recherche (ANR), n. d.)—thus collectively contributing to the coherence and overall reach of prevention research in France. Moreover, as several cancer risk factors are shared with other diseases—for example, cardiovascular diseases in the case of tobacco and alcohol—prevention research efforts targeting these factors and funded in fields other than cancer by other organizations complement those supported by INCa. In addition, national funding for public research infrastructure and personnel, which is not reflected in the budgets allocated by funding agencies, must also be taken into account.

The implementation of the Ten-Year Cancer Control Strategy 2021–2030 (Institut national du cancer (INCa), 2021) has significantly contributed to strengthening the deployment of PHIR through concrete actions and priorities, for which the contribution of PHIR—and more broadly of the social sciences and humanities—is expected. These include personalized prevention approaches, improved access to screening, the promotion of health-enhancing behaviours, and the development of supportive care, among others. At the same time, the participation of citizens, patients, workers, and more broadly the beneficiaries of interventions has become increasingly widespread and valued in scientific and institutional discourse. In this regard, INCa has launched a health democracy committee. However, within research settings, stakeholder participation remains a key challenge from methodological, ethical, and organizational perspectives. It was in this context that INCa organized, on December 12–13, 2023, the 6th International Francophone Conference entitled: “*Intervening and acting with and for citizens, patients, and workers: Contributions of population health intervention research in the field of cancer*.”

The contributions brought together in this supplement examine these issues with rigour and nuance. They show that “doing with and for” is not merely a methodological principle, but entails a redefinition of relationships among researchers, professionals, field actors, patients, and citizens. This participatory dynamic invites us to move beyond traditional silos between disciplinary fields, living environments, and social statuses, recognizing that patients are also citizens and workers, and vice versa.

A first set of contributions explores the conceptual, methodological, and theoretical foundations of intervention research, with a focus on the co-construction of interventions, knowledge capitalization, and frameworks for thinking about evaluation and transferability (Villeval et al.; Beuriot et al.).

A second axis addresses cancer prevention and early detection interventions, particularly in workplace settings or among specific populations (Rollin et al.; Crasset et al.; Hunsmann et al.).

Other contributions highlight participatory and inclusive approaches, examining the involvement of patients, the public, or vulnerable groups in knowledge production and health learning processes (Bauquier et al.; Oustric et al.; Satgé et al.).

Finally, a fourth axis examines the conditions for implementation, scaling up, and sustainability of complex interventions, whether related to smoking cessation, screening, improvement of care experiences, or social and economic support for people in precarious situations (Stevens et al.; Burtin et al.; Pérignon et al.; Pomey et al.).

As health and social challenges continue to grow in complexity, intervention research appears more essential than ever as a tool for acting with and for populations, in support of a cancer control effort that is effective, equitable, and sustainable. However, at a time when the international context requires each expenditure to be justified, it is crucial that every research domain demonstrate the role it has played in collective progress. This applies, for example, to the reduction of daily smoking, where analysis—including health economic evaluation—of the respective contributions of cognitive-behavioural efforts and regulatory interventions (pricing, bans, substitution strategies) cannot be overlooked.

This supplement therefore aims to spark interest among all stakeholders involved in intervention research, in order to encourage the development of new collaborations and initiatives.

## Footnotes

1 Le programme Tabado a été lancé en 2018 et accompagne gratuitement chaque année plus de 83.000 jeunes apprentis et lycéens professionnels dans l’arrêt du tabac, en proposant des interventions en établissement (réunions collectives et suivi individuel par des professionnels). Ce programme est coordonné au niveau national par l’INCa et bénéficie d’un financement du Fonds de lutte contre les addictions (FLCA).

2 The Tabado programme was launched in 2018 and provides free support each year to more than 83,000 young apprentices and students in vocational high schools to help them quit smoking. It offers school-based interventions, including group sessions and individual follow-up by trained professionals. The programme is coordinated at the national level by the French National Cancer Institute (INCa) and is funded by the Fund for th
